# Urgent referral for suspected CNS cancer: which clinical features are associated with a positive predictive value of 3 % or more?

**DOI:** 10.1186/s12883-016-0677-1

**Published:** 2016-08-26

**Authors:** Hasan Raza Mohammad, Jeremy Boardman, Laura Howell, Roger J. Mills, Hedley C. A. Emsley

**Affiliations:** 1 Department of Neurology, Royal Preston Hospital, Sharoe Green Lane, Fulwood, Preston PR2 9HT UK; 2 Faculty of Biology, Medicine and Health, University of Manchester, Oxford Road, Manchester, M13 9PL UK; 3 College of Health and Wellbeing, University of Central Lancashire, Fylde Road, Preston, PR1 2HE UK

**Keywords:** CNS cancer, Retrospective study, Two-week referral, Positive predictive value, NICE guidance

## Abstract

**Background:**

Urgent referral for suspected central nervous system (CNS) cancer is recommended, but little analysis of the referral criteria diagnostic performance has been conducted. New 2015 NICE guidance recommends direct brain imaging for patients with symptoms with positive predictive values (PPV) of 3 %, but further guidance is needed.

**Methods:**

A 12-month retrospective evaluation of 393 patients referred under previous 2005 NICE 2-week rule criteria was conducted. Analysis was based on the three groups of symptoms forming the referral criteria, (1) CNS symptoms, (2) recent onset headaches, (3) rapidly progressive subacute focal deficit/cognitive/behavioural/personality change. Comparison was made with neuroimaging findings.

**Results:**

Twelve (3.1 %) of 383 patients who attended clinic had CNS cancer suggesting the combination of clinical judgement and application of 2005 criteria matched the 2015 guideline’s PPV threshold. PPVs for the three groups of symptoms were (1) 4.1 % (95 % CIs 2.0 to 7.4 %), (2) 1.2 % (0.1 to 4.3 %) and (3) 3.7 % (0.1 to 19.0 %). Sensitivities were (1) 83.3 % (95 % CIs 51.6 to 97.9 %), (2) 16.7 % (2.1 to 48.4 %), and (3) 8.3 % (0.2 to 38.5 %); specificities were (1) 37.2 % (32.3 to 42.3 %), (2) 55.5 % (50.3 to 60.7 %) and (3) 93.0 % (89.9 to 95.4 %). Of 288 patients who underwent neuroimaging, 59 (20.5 %) had incidental findings, most commonly cerebrovascular disease.

**Conclusions:**

The 2015 guidance is less prescriptive than previous criteria making clinical judgement more important. CNS symptoms had greatest sensitivity, while PPVs for CNS symptoms and rapidly progressive subacute deficit/cognitive/behavioural/personality change were closest to 3 %. Recent onset headaches had the lowest sensitivity and PPV.

**Electronic supplementary material:**

The online version of this article (doi:10.1186/s12883-016-0677-1) contains supplementary material, which is available to authorized users.

## Background

The 1990s saw rising waiting times in the United Kingdom (UK) for patients undergoing investigation of suspected cancer, including suspected central nervous system (CNS) cancer. This prompted the Department of Health to introduce guidelines in 2000 for referral, with structured pathways and a waiting time target of 2 weeks [1, 2]. The referral guidelines for suspected cancer were revised in 2005 [3] and completely overhauled in 2015 [4] because of concerns that cancer survival in the UK is lower than in other developed countries. The latest guidelines for adults with suspected CNS cancer (Table 1), which advocate direct referral for brain imaging to be performed within 2 weeks, represent a substantial shift from the 2005 guidelines (Table 2) which comprised clinical criteria based on groups of symptoms for urgent outpatient referral (typically to neurology) to be seen within 2 weeks, or for referral to be considered.

**Table 1:** (From 2015 guidelines [4])

Consider an urgent direct access MRI scan of the brain (or CT scan if MRI is contraindicated) (to be performed within 2 weeks) to assess for brain or central nervous system cancer in adults with progressive, sub-acute loss of central neurological function

**Table 2:** (From 2005 guidelines [3])

Refer urgently patients with:
• symptoms related to the CNS, including: - progressive neurological deficit - new-onset seizures - headaches - mental changes - cranial nerve palsy - unilateral sensorineural deafness in whom a brain tumour is suspected • headaches of recent onset accompanied by features suggestive of raised intracranial pressure, for example: - vomiting - drowsiness - posture-related headache - pulse-synchronous tinnitus or by other focal or non-focal neurological symptoms, for example blackout, change in personality or memory • a new, qualitatively different, unexplained headache that becomes progressively severe • suspected recent-onset seizures (refer to neurologist)
Consider urgent referral (to an appropriate specialist) in patients with rapid progression of:
• subacute focal neurological deficit • unexplained cognitive impairment, behavioural disturbance or slowness, or a combination of these • personality changes confirmed by a witness and for which there is no reasonable explanation even in the absence of other symptoms and signs of a brain tumour

Currently, the extent of implementation of the 2015 guidelines for suspected CNS cancer is somewhat variable, with gradual transition being expected from the 2005 to the 2015 guidelines while the implications for clinical practice, including referral pathways and impact on imaging and reporting capacity etc., are understood.

The 2015 guidelines advise that adults with clinical features that are associated with a positive predictive value (PPV) of 3 % or more for CNS cancer should be referred urgently for investigation [4]. The new guidelines are much less prescriptive in their wording, particularly, in respect of which clinical features might be the most relevant. Relatively little is known about the diagnostic performance of the 2005 referral criteria, or diagnosis rate of CNS cancer among patients referred using those criteria [5]. The likely effects of the 2015 guidelines upon referral behaviour and the implications for direct access imaging requests is, to all intents and purposes, unknown. An improved understanding of the diagnostic performance of the 2005 criteria and which clinical features are relevant in determining whether there is a 3 % or greater likelihood of CNS cancer will surely be helpful. In addition, relatively little is known about the implications for patients and clinical pathways upon the identification of incidental findings when imaging is being requested directly from primary care [6].

We have undertaken a retrospective study of patients referred under the ‘2 week rule’ for suspected CNS cancer according to the 2005 guidelines over a 12 month period. We have analysed (1) the diagnostic performance of the 2005 criteria, with a clinical and radiological diagnosis of CNS cancer as the primary outcome, (2) the symptom frequencies amongst all referred patients and those with CNS cancer, and (3) incidental findings.

## Methods

### Data extraction and validation

Routine clinical data were extracted from referral letters, clinic letters and imaging reports for all patients referred under the ‘2 week rule’ for suspected CNS cancer to the regional neurology service based at the Royal Preston Hospital (serving a population of approximately 1.6 million) between 1^st^ June 2012 and 31^st^ May 2013. Data were extracted by one junior doctor (HM, who was an undergraduate at the time of data collection), and were independently validated by a second junior doctor (JB) working in neurology. One year after data collection, all patients’ records were reviewed to determine whether any other visits/imaging had occurred.

### Classification of 2005 referral criteria and analysis

Referral criteria (2005 criteria) were grouped as follows: group 1 – symptoms related to the CNS (all new-onset or recent-onset seizures were included in this group), group 2 – headaches of recent onset accompanied by features suggestive of raised intracranial pressure (ICP), and group 3 – rapidly progressive subacute focal deficit/cognitive/behavioural or personality change. Presenting symptoms were classified into one of these three groups. The primary outcome was the presence/absence of CNS cancer on the basis of clinical assessment and, where applicable, neuroimaging findings (CT brain/MRI brain). Sensitivity, specificity, positive and negative predictive values were calculated for each symptom group.

### Statistical methods

Statistical analysis was performed using Stata (StataCorp. 2013. *Stata Statistical Software: Release 13*. College Station, TX: StataCorp LP) and StatsDirect (StatsDirect Ltd. StatsDirect statistical software. http://www.statsdirect.com. England: StatsDirect Ltd. 2013).

Presenting symptoms were reported for all referrals and by CNS cancer diagnosis using frequencies and percentages. Comparisons of presenting symptoms by CNS cancer diagnosis were performed using the Fisher’s exact test. To avoid multiple testing, comparisons were made only for the overall presenting symptom groups: symptoms related to CNS cancer, headaches of recent onset accompanied by features suggestive of raised intracranial pressure and consider urgent referral. The significance threshold was set at *p* ≤ 0.05. Measures of diagnostic performance, sensitivity, specificity, positive predictive value and negative predictive value were reported for each of the symptom groups based on participants who were referred and attended clinic. Diagnosis of CNS cancer was based on clinical decision and radiological findings. Please see Additional file 1: Appendix for raw data calculations.

## Results

**Fig. 1:**
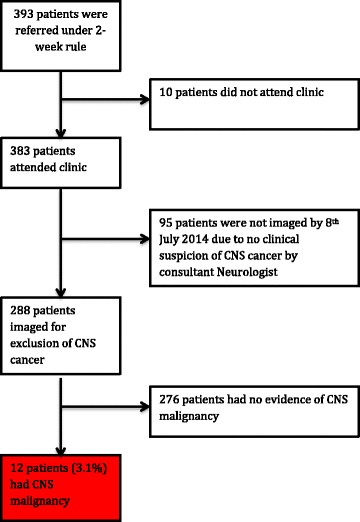
Flow chart of patients recruited in the study

Between 1^st^ June 2012 and 31^st^ May 2013, 393 adult patients were referred under the ‘2 week rule’ for suspected CNS cancer. Ten patients did not attend their appointment or were seen at another hospital. Three hundred and eighty-three patients attended clinic, of whom 95 did not undergo neuroimaging (and did not undergo imaging by July 2014) on account of the neurologist considering there to be no clinical suspicion of CNS cancer and no other indication for scanning. Two hundred and eighty eight patients underwent neuroimaging (Fig. 1).

### CNS cancer diagnoses

Twelve patients were found to have CNS cancer. This constitutes 3.1 % of the total number of referred patients who attended their appointment and 4.2 % of patients who underwent imaging. Histopathological diagnoses were grade IV glioblastoma in 4 cases, lung cancer metastases in 2 cases, and anaplastic oligoastrocytoma in 1 case. No biopsies were available in the remaining 5 cases. Radiological and clinical diagnoses of these 5 cases were: cystic glioma, metastases from unknown source in parasagittal region, lung metastases in posterior corpus callosum, non-small cell carcinoma metastases in right parietal tissue and C2 vertebral body metastases without frank spinal cord compression.

### Diagnostic performance of 2005 referral criteria

The frequency of presenting symptoms and symptom groups are shown in Table 3. Two hundred and forty-three patients were referred with group 1 symptoms, 167 with group 2 symptoms, and 27 with group 3 symptoms. For group 1 (symptoms related to the CNS), the sensitivity was 83.3 % (95 % CI 51.6 to 97.9 %), specificity 37.2 % (95 % CI 32.3 to 42.3 %), PPV 4.1 % (95 % CI 2.0 to 7.4 %) and negative predictive value (NPV) 98.6 % (95 % CI 94.9 to 99.8 %). For group 2 (headaches of recent onset accompanied by features suggestive of raised intracranial pressure), the sensitivity was 16.7 % (95 % CI 2.1 to 48.4 %), specificity 55.5 % (95 % CI 50.3 to 60.7 %), PPV 1.2 % (95 % CI 0.1 to 4.3 %) and NPV 95.4 % (95 % CI 91.7 to 97.8 %). For group 3 (rapidly progressive subacute focal deficit/cognitive/behavioural or personality change), the sensitivity was 8.3 % (95 % CI 0.2 to 38.5 %), specificity 93.0 % (95 % CI 89.9 to 95.4 %), PPV 3.7 % (95 % CI 0.1 to 19.0 %) and NPV 96.9 % (95 % CI 94.5 to 98.4 %).

**Table 3:** The prevalence of symptoms in referrals under the 2-week rule for suspected CNS cancer

Presenting symptom	All referrals (*n* = 383)	No CNS cancer (*n* = 371)	CNS cancer (*n* = 12)	*p*
Symptoms related to the CNS	243 (63.4)	233 (62.8)	10 (83.3)	0.224
Progressive neurological deficit	30 (7.8)	27 (7.3)	3 (25.0)	
New-onset seizures	41 (10.7)	39 (10.5)	2 (16.7)	
Headaches	173 (45.2)	168 (45.3)	5 (41.7)	
Mental changes	21 (5.5)	19 (5.1)	2 (16.7)	
Cranial nerve palsy	19 (5.0)	18 (4.9)	1 (8.3)	
Unilateral sensorineural deafness	10 (2.6)	10 (2.7)	0 (0.0)	
Headaches of recent onset accompanied by features suggestive of raised intracranial pressure	167 (43.6)	165 (44.5)	2 (16.7)	0.075
Vomiting	28 (7.3)	28 (7.5)	0 (0.0)	
Drowsiness	23 (6.0)	23 (6.2)	0 (0.0)	
Posture-related headache	68 (17.8)	67 (18.1)	1 (8.3)	
Pulse-synchronous tinnitus	3 (0.8)	3 (0.8)	0 (0.0)	
Other focal/non-focal neurological problems	71 (18.5)	70 (18.9)	1 (8.3)	
New, qualitatively different, unexplained headache that becomes progressively severe	43 (11.2)	43 (11.6)	0 (0.0)	
Consider urgent referral - rapidly progressive subacute focal deficit/cognitive/behavioural or personality change	27 (7.0)	26 (7.0)	1 (8.3)	0.590
Subacute focal neurological deficit	7 (1.8)	6 (1.6)	1 (8.3)	
Unexplained cognitive impairment/behavioural disturbance or slowness, or a combination of these	17 (4.4)	17 (4.6)	0 (0.0)	
Personality changes	9 (2.3)	9 (2.4)	0 (0.0)	

*p*-value derived from Fisher’s Exact Test comparing presence of symptom groups between patients with and without confirmed CNS cancer

### Incidental findings

Of 288 patients who underwent neuroimaging, 59 (20.5 %) were found to have incidental findings (Table 4). Cerebrovascular disease (11.1 %), degenerative spine disease (3.5 %) and sinus disease (3.1 %) were the most frequent incidental findings.

**Table 4:** Summary of incidental findings on neuroimaging

Incidental finding	Number of patients (%)
Benign cystic lesion	5 (1.7 %)
Cerebrovascular disease	32 (11.1 %)
*Small vessel disease only*	25 (8.7 %)
*Large artery disease only*	5 (1.7 %)
*Mixed small vessel and large artery disease*	2 (0.7 %)
Degenerative spine disease	10 (3.5 %)
*Cervical*	9 (3.1 %)
*Lumbar*	1 (0.3 %)
CNS demyelination	3 (1.0 %)
Sinus disease	9 (3.1 %)

## Discussion

This study, to our knowledge, is the first to consider the implications of the recently revised NICE guidelines for suspected CNS cancer, and specifically which clinical features are associated with a PPV of 3 % or more by analysing the diagnostic performance of previous referral criteria [5].

Anecdotally, there can appear to be excessive ‘2 week rule’ referrals for suspected CNS cancer to neurology clinics. With the 2015 guidelines advocating direct referral for imaging, fewer patients with suspected CNS cancer might be expected to attend neurology clinics, although the identification of incidental findings will inevitably also have implications for clinical care. In the present study, ‘2 week rule’ referrals constituted approximately 3 % of the total number of new outpatient referrals to the regional neurology service (ca. 12,000). The overall CNS cancer diagnosis rate among the ‘2 week rule’ referral population was 3.1 % (i.e. 12 CNS cancer diagnoses among 383 patients who were referred by this route and who attended clinic). This finding would appear to suggest that intriguingly, through clinical judgement and the application of the 2005 referral criteria, there was a pattern of referral behaviour for suspected CNS cancer matching the PPV threshold of 3 % at which patients should be urgently referred, according to the 2015 guidelines [4].

Now that the referral criteria are much less prescriptive, referrers will, more than ever, have to employ clinical judgement when considering referral. But which clinical features would suggest a PPV of 3 % or more? Headache often tends to prompt concerns in the patient and the referrer about the possibility of CNS cancer but performs very poorly as a predictor [7]. Probably undue emphasis is placed on headache *per se*, and the findings in the present study support the usual view that as a single symptom it does tend to be a poor discriminator with respect to the presence/absence of CNS cancer. Nonetheless, headache accounts for 4.4 % of primary care consultations and up to 30 % of outpatient neurology referrals in the UK [8, 9]. However, the current analysis highlights focal deficits (subacute or progressive), new-onset seizures, or cognitive/behavioural/personality changes, as being more strongly predictive of CNS cancer in the appropriate clinical context. New-onset seizures in particular, whether focal or secondary generalised, can be an important early manifestation of a brain tumour. In a previous study of clinical features and the risk of primary brain tumours, in which new-onset epilepsy had an overall risk of 1.2 %, rising to 2.3 % if the patient was >60 years of age, in marked contrast to the risk with headache, which was associated with a risk of less than 1 in 1000 [10].

It should be noted that diagnostic performance of all three symptom groups in this study was poor by comparison with usual expectations for a good diagnostic test which would have both a high sensitivity and specificity (both around 90.0 %) [11]. Headaches of recent onset accompanied by features suggestive of raised ICP were actually less frequent among patients found to have CNS cancer than among the total referral population. Potentially this suggests difficulties in clinical recognition of features of raised ICP. Uncertainty among referrers over headache diagnosis has certainly been reported previously [12].

Bypassing a neurology clinical opinion *en route* to brain imaging may raise some issues in patient management, particularly with respect to relative lack of a detailed neurological assessment which, at least anecdotally, can be helpful for contextualising incidental findings. Impact of the NICE guidance with respect to imaging and reporting capacity is uncertain. An international report published by the Organisation for Economic Cooperation and Development (OECD) found that the UK had fewer magnetic resonance imaging (MRI) scanners than almost any other Western country including developing countries such as Turkey and Slovakia [13]. Out of 32 countries in the OECD the UK stands 26^th^. For computerised tomography (CT) scanning, the UK is 30^th^ of 32 [13].

Brain scans are preferably reported by a neuroradiologist, which creates issues of hospital’s reporting capacity.

Implementation of the 2015 NICE criteria also needs to take into account the frequent identification of incidental findings. A systematic review and meta-analysis reported incidental findings of 2.7 % from 19,559 participants [14]. The study suggested that at the very least clinicians should counsel patients about the chance of incidental findings prior to requesting a scan and that a mechanism for their management would need to be implemented [14]. There is considerable uncertainty surrounding the management of some incidental findings on brain imaging, including balancing risk/benefit of intervention for intracranial aneurysms [15, 16], unruptured arteriovenous malformations [17], low grade glioma [18] and arachnoid cysts [14]. There is little evidence to guide the management of incidental radiological cerebrovascular disease. This lack of certainty can create significant patient anxiety, lead to additional referrals/investigations, sometimes with significant implications for the patient [19–21]. It seems wise for pre-imaging counselling to make reference not only to the possibility of incidental findings but also uncertainty in their management.

By necessity, given the study design, the calculation of PPVs and NPVs is based on the referral population. This does limit the extent to which these values are directly applicable to the total population (i.e. including an unknown number of unreferred patients with relevant symptoms). Clearly, the balance of positive and negative imaging findings among unreferred patients is also unknown.

## Conclusions

The new 2015 guidance is less prescriptive than previous CNS cancer referral criteria making clinical judgement even more important. Symptoms related to the CNS had the greatest sensitivity, while PPVs for symptoms related to the CNS and rapidly progressive subacute deficit/cognitive/behavioural/personality change were closest to the NICE referral figure of 3 %. Headaches of recent onset had the lowest sensitivity and PPV; diagnostic performance with respect to sensitivity and specificity was poor for all three symptom groups. The frequent occurrence of incidental findings also needs to be taken into account when requesting imaging and planning services.

## Additional file

Additional file 1:
**Appendix.** Raw data calculations. (DOCX 31 kb)

## Abbreviations

CNS: Central nervous system
CT: Computerised tomography
ICP: Intracranial pressure
MRI: Magnetic resonance imaging
NPV: Negative predictive value
NHS: National health service
OECD: Organisation for Economic Cooperation and Development
PPV: Positive predictive value
UK: United Kingdom
