# Supplementary material for: Urgent referral for suspected CNS cancer: which clinical features are associated with a positive predictive value of 3 % or more?
Source: BMC Neurol. 2016 Aug 26;16(1):152. doi: 10.1186/s12883-016-0677-1 (PMC5000444; doi:10.1186/s12883-016-0677-1)
Supplement: Additional file 1: — Appendix. Raw data calculations. (DOCX 31 kb) [file 12883_2016_677_MOESM1_ESM.docx]

Raw data calculations

Table 1: Frequency of presenting symptoms among all patients referred and patients with CNS cancer

| **Presenting symptom**  **N (%)** | **All referrals (n=383)** | **No CNS cancer (n=371)** | **CNS cancer (n=12)** | **P-value^1^** |
| --- | --- | --- | --- | --- |
| **Symptoms related to the CNS** | 243 (63.4) | 233 (62.8) | 10 (83.3) | 0.224 |
| Progressive neurological deficit | 30 (7.8) | 27 (7.3) | 3 (25.0) |  |
| New-onset seizures | 41 (10.7) | 39 (10.5) | 2 (16.7) |  |
| Headaches | 173 (45.2) | 168 (45.3) | 5 (41.7) |  |
| Mental changes | 21 (5.5) | 19 (5.1) | 2 (16.7) |  |
| Cranial nerve palsy | 19 (5.0) | 18 (4.9) | 1 (8.3) |  |
| Unilateral sensorineural deafness | 10 (2.6) | 10 (2.7) | 0 (0.0) |  |
| **Headaches of recent onset accompanied by features suggestive of raised intracranial pressure** | 167 (43.6) | 165 (44.5) | 2 (16.7) | 0.075 |
| Vomiting | 28 (7.3) | 28 (7.5) | 0 (0.0) |  |
| Drowsiness | 23 (6.0) | 23 (6.2) | 0 (0.0) |  |
| Posture-related headache | 68 (17.8) | 67 (18.1) | 1 (8.3) |  |
| Pulse-synchronous tinnitus | 3 (0.8) | 3 (0.8) | 0 (0.0) |  |
| Other focal/non-focal neurological problems | 71 (18.5) | 70 (18.9) | 1 (8.3) |  |
| New, qualitatively different, unexplained headache that becomes progressively severe | 43 (11.2) | 43 (11.6) | 0 (0.0) |  |
| **Consider urgent referral** | 27 (7.0) | 26 (7.0) | 1 (8.3) | 0.590 |
| Subacute focal neurological deficit | 7 (1.8) | 6 (1.6) | 1 (8.3) |  |
| Unexplained cognitive impairment/behavioural disturbance or slowness, or a combination of these | 17 (4.4) | 17 (4.6) | 0 (0.0) |  |
| Personality changes | 9 (2.3) | 9 (2.4) | 0 (0.0) |  |

^1^ P-value produced from Fisher’s exact test comparing presence of symptom groups with CNS cancer diagnosis

Table 2: Frequency of symptom groups by CNS cancer

| **Symptoms**  **N (%)** | **No CNS cancer**  **(n=371)** | **CNS cancer**  **(n=12)** |
| --- | --- | --- |
| No symptoms | 84 (22.6) | 1 (8.3) |
| At least 1 symptom group | 159 (42.9) | 9 (75.0) |
| At least 2 symptom groups | 119 (32.1) | 2 (16.7) |
| All 3 symptom groups | 9 (2.4) | 0 (0.0) |

**Symptoms relating to CNS cancer**

Sensitivity = 10/12 = 83.3% with a 95% confidence interval 51.6% to 97.9%.

Specificity = 138/371 = 37.2% with a 95% confidence interval 32.3% to 42.3%.

Positive Predictive Value (PPV) = 4.1% with a 95% confidence interval 2.0% to 7.4%.

Negative Predictive Value (NPV) = 98.6% with a 95% confidence interval 94.9% to 99.8%.

**Headaches of recent onset**

Sensitivity = 2/12 = 16.7% with a 95% confidence interval 2.1% to 48.4%.

Specificity = 206/371 = 55.5% with a 95% confidence interval 50.3% to 60.7%.

Positive Predictive Value (PPV) = 2/167 = 1.2% with a 95% confidence interval 0.1% to 4.3%.

Negative Predictive Value (NPV) = 206/216 = 95.4% with a 95% confidence interval 91.7% to 97.8%.

**Consider urgent referral**

Sensitivity = 1/12 = 8.3% with a 95% confidence interval 0.2% to 38.5%.

Specificity = 345/371 = 93.0% with a 95% confidence interval 89.9% to 95.4%.

Positive Predictive Value (PPV) = 1/27 = 3.7% with a 95% confidence interval 0.1% to 19.0%.

Negative Predictive Value (NPV) = 345/356 = 96.9% with a 95% confidence interval 94.5% to 98.4%.

**Flow chart**

393 patients referred

10 patients did not attend and were not imaged.

383 patients attended clinic

95 patients were not imaged

288 patients were imaged

12 patients had malignancy

**Statistical methods**

Statistical analysis was performed using Stata (StataCorp. 2013. *Stata Statistical Software: Release 13*. College Station, TX: StataCorp LP) and StatsDirect (StatsDirect Ltd. StatsDirect statistical software. <http://www.statsdirect.com>. England: StatsDirect Ltd. 2013).

Presenting symptoms were reported for all referrals and by CNS cancer diagnosis using frequencies and percentages. Comparisons of presenting symptoms by CNS cancer diagnosis were performed using the Fisher’s exact test. To avoid multiple testing, comparisons were made only for the overall presenting symptom groups: symptoms related to CNS cancer, headaches of recent onset accompanied by features suggestive of raised intracranial pressure and consider urgent referral. A P < 0.05 determined significance. Measures of diagnostic success, sensitivity, specificity, positive predictive value and negative predictive value were reported for each of the symptom groups based on participants who were referred and attended clinic. Diagnosis of CNS cancer was based on clinical decision and radiological findings.
